# Exposure of Monocytic Cells to Lipopolysaccharide Induces Coordinated Endotoxin Tolerance, Mitochondrial Biogenesis, Mitophagy, and Antioxidant Defenses

**DOI:** 10.3389/fimmu.2018.02217

**Published:** 2018-09-27

**Authors:** John D. Widdrington, Aurora Gomez-Duran, Angela Pyle, Marie-Helene Ruchaud-Sparagano, Jonathan Scott, Simon V. Baudouin, Anthony J. Rostron, Penny E. Lovat, Patrick F. Chinnery, A. John Simpson

**Affiliations:** ^1^Institute of Cellular Medicine, Newcastle University, Newcastle upon Tyne, United Kingdom; ^2^Institute of Genetic Medicine, Newcastle University, Newcastle upon Tyne, United Kingdom; ^3^MRC Mitochondrial Biology Unit, Cambridge Biomedical Campus, Cambridge, United Kingdom; ^4^Department of Clinical Neurosciences, University of Cambridge, Cambridge, United Kingdom; ^5^Department of Anaesthesia, Royal Victoria Infirmary, Newcastle upon Tyne, United Kingdom

**Keywords:** inflammation, endotoxin tolerance, mitochondria, mtDNA, mitophagy, mitochondrial biogenesis, antioxidants

## Abstract

In order to limit the adverse effects of excessive inflammation, anti-inflammatory responses are stimulated at an early stage of an infection, but during sepsis these can lead to deactivation of immune cells including monocytes. In addition, there is emerging evidence that the up-regulation of mitochondrial quality control mechanisms, including mitochondrial biogenesis and mitophagy, is important during the recovery from sepsis and inflammation. We aimed to describe the relationship between the compensatory immune and mitochondrial responses that are triggered following exposure to an inflammatory stimulus in human monocytic cells. Incubation with lipopolysaccharide resulted in a change in the immune phenotype of THP-1 cells consistent with the induction of endotoxin tolerance, similar to that seen in deactivated septic monocytes. After exposure to LPS there was also early evidence of oxidative stress, which resolved in association with the induction of antioxidant defenses and the stimulation of mitochondrial degradation through mitophagy. This was compensated by a parallel up-regulation of mitochondrial biogenesis that resulted in an overall increase in mitochondrial respiratory activity. These observations improve our understanding of the normal homeostatic responses that limit the adverse cellular effects of unregulated inflammation, and which may become ineffective when an infection causes sepsis.

## Introduction

During sepsis an infection triggers a systemic inflammatory response, leading to organ dysfunction, shock and a significant risk of mortality (1). The clinical outcome of sepsis appears to be determined by the initial host inflammatory response to the infection and the subsequent compensatory mechanisms leading to the resolution of this inflammation (2). The dysregulation of any of these processes may result in complications. In particular, an excessive or prolonged immune deactivation phase later in the sepsis illness leads to a vulnerability to nosocomial infections and increased mortality (3). Deactivation of blood monocytes, key innate immune cells, appears to be particularly important during this sepsis-induced immune deactivation but the mechanisms underlying this process are not well understood (4–6).

There is increasing evidence that impaired cellular respiration due to mitochondrial dysfunction during sepsis is associated with adverse clinical outcomes and may lead to impaired monocyte functions (7–9). Mitochondria are organelles with several critical cellular functions, particularly the generation of cellular energy at five enzyme complexes on the inner mitochondrial membrane during oxidative phosphorylation (OXPHOS) (10). The majority of mitochondrial constituents are encoded on the nuclear genome but mitochondria also contain circular mitochondrial DNA (mtDNA) with genes encoding 13 essential OXPHOS complex subunits (11). During sepsis mitochondria may become damaged or dysfunctional, leading to mtDNA depletion, impaired cellular respiration, and cell death (12, 13). The persistent presence of dysfunctional mitochondria can also lead to oxidative stress, as mitochondria are the main source of reactive oxygen species through the leakage of electrons during OXPHOS, and act as a potent stimulus for ongoing inflammation (14, 15).

The adverse effects of inflammation on mitochondria can be abrogated by several mechanisms. These include the induction of anti-inflammatory responses and antioxidant defenses, maintenance of mitochondrial integrity through the selective removal of dysfunctional mitochondria (mitophagy), and the generation of new organelles to replace them (mitochondrial biogenesis) (16, 17). However, the integration of these compensatory responses, and the interaction between mitochondria and immunity in monocytic cells following an inflammatory insult, are not well understood. In order to study these processes we assessed mitochondrial functions, biogenesis, and mitophagy in a time course model of endotoxin tolerance, a process whereby repeated exposure to lipopolysaccharide (LPS) from Gram negative bacteria leads to a change in immune phenotype similar to that seen in deactivated blood monocytes (18).

Here we show that there is a reversible induction of antioxidant defenses, mitophagy, and mitochondrial biogenesis in THP-1 cells rendered endotoxin tolerant following exposure to LPS, leading to a maintenance of cellular viability and respiration. These findings suggest that these processes are vital to cellular recovery following an inflammatory insult and that dysregulation of these compensatory mechanisms may contribute to adverse outcomes when an infection causes sepsis.

## Material and methods

### THP-1 cell culture and reagents

**Figure 1:**
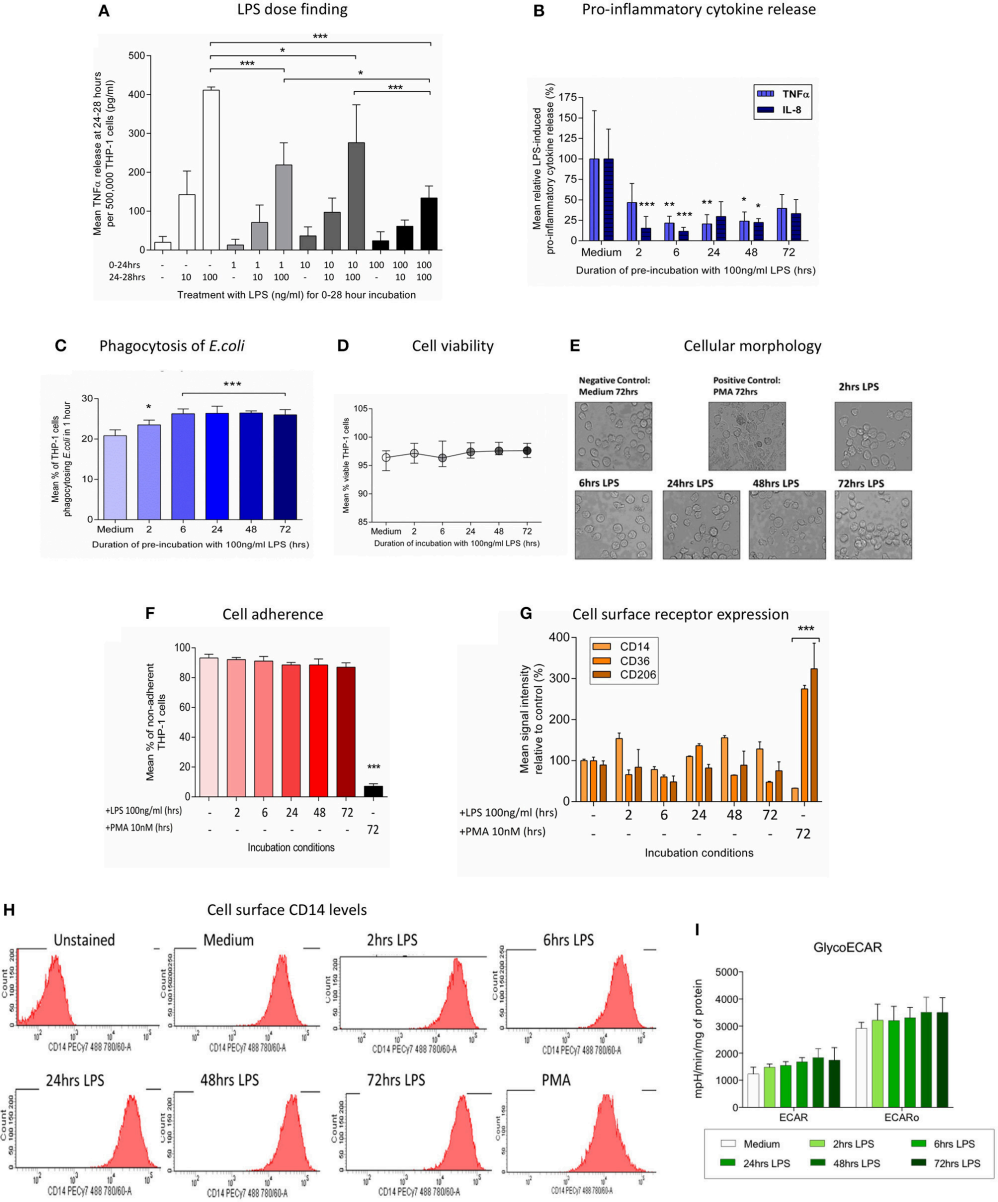
Pre-incubation of THP-1 cells with LPS results in a change in immune phenotype consistent with endotoxin tolerance. THP-1 cells were pre-incubated with LPS (100 ng/ml) for 0–72 h and the ability to respond to a second inflammatory stimulus was then determined. **(A)** THP-1 cells were pre-incubated with 1, 10, or 100 ng/ml LPS for 24 h before measuring release of TNFα during a further 4-h exposure to a second stimulus of 10 or 100 ng/ml LPS by ELISA. **(B)**The release of pro-inflammatory TNFα and IL-8 in response to a second exposure to LPS (100 ng/ml) was measured by ELISA (*n* = 6). **(C)** Phagocytosis of fluorescein-labeled *Escherichia coli* (*E.coli*) in 1 h was measured by flow cytometry (*n* = 4). **(D)** Cell viability was determined by measuring the proportion of cells excluding propidium iodide following incubation with LPS (100 ng/ml) for 0–72 h (*n* = 4). **(E)** THP-1 cells were incubated with LPS (100 ng/ml) for 0–72 h before measuring macrophage differentiation in comparison to a positive control of cells treated with 10 nM PMA for 72 h. Representative images of THP-1 cell morphology on inversion light microscope at 40x magnifications. **(F)** THP-1 cell adherence to a 6 well plate was determined by cell counts before and after removal of adherent cells using a cell scraper (*n* = 3). **(G)** Flow cytometry was used to determine the relative signal intensity for the expression of the markers of macrophage differentiation CD14, CD36, and CD206 compared to the mean in the medium control (*n* = 3) **(H)** Representative histograms indicating the level of cell surface CD14 expression measured by flow cytometry. **(I)** Extracellular acidification rate with and without olygomycin following exposure to LPS (100 ng/ml) for 0–72 h as determined using the Seahorse XF96^e^ extracellular flux analyser (*n* = 5). All data are presented as mean ± standard deviation with the values in panel a expressed as relative cytokine release compared to the mean in the medium control; *p < 0.05, **p < 0.01, ***p < 0.001.

All reagents were obtained from ThermoFisher Scientific (Waltham, MA, USA) unless otherwise stated. THP-1 cells (ATCC®TIB-202TM) were kindly provided by Dr John Taylor's laboratory, Newcastle University. The cells were maintained at a concentration of < 1 × 10^6^ cells/ml in RPMI 1640 medium supplemented with 10% fetal calf serum (FCS) and contamination with Mycoplasma was periodically excluded. In all experiments 1 × 10^6^ THP-1 cells were incubated in 25 cm^3^ tissue flasks containing 5 ml growth medium to which LPS (100 ng/ml) from *Escherichia coli* O26/B6 (Sigma-Aldrich, St Louis, MO, USA) was added either 72 (*t* = 0 h), 48 (*t* = 24 h), 24 (*t* = 48 h), 6 (*t* = 66 h), or 2 (*t* = 70 h) h prior to the end of a 72 h pre-incubation period. After this pre-incubation the THP-1 cells were then pelleted, washed with PBS and re-suspended in fresh medium before comparing immune and mitochondrial functions to those in control cells pre-incubated for the previous 72 h in growth medium without LPS. The dose of LPS used in this model was selected on the basis of dose-finding experiments assessing the optimal induction of endotoxin tolerance (Figure 1A). *E. coli* O26/B6 LPS was chosen as we and others have shown inhalation to produce reproducible inflammation in human volunteers (19, 20).

### Monocyte isolation and culture

Whole blood was obtained from 5 healthy volunteers (ethical approval was obtained from the relevant Research Ethics Committee and all volunteers provided informed, written consent). Human peripheral blood mononuclear cells (PBMCs) were extracted from the whole blood using dextran (Pharmacosmos, Holbaek, Denmark) sedimentation and Percoll (GE Healthcare Biosciences, Little Charlfort, UK) density-gradient centrifugation (21). Using the MACS Monocyte Isolation Kit II, MS columns and the Mini-MACS Separator (all Miltenyi Biotec, Auburn, CA, USA) monocytes were isolated from the PBMC fraction by negative selection. The purity of isolated monocytes was confirmed at >95% using morphological assessment following cytospin with Giemsa staining. The monocytes were re-suspended in IMDM medium supplemented with 10% autologous human serum and cultured with or without 10 ng/ml LPS for 24 h before measuring immune and mitochondrial functions.

### Detection of cytokine production

2.5 × 10^5^ THP-1 cells or 1 × 10^5^ monocytes in 500 μl growth medium per well were seeded onto a 24 well plate (Grenier Bio-one, Stonehouse, UK) and incubated for 4 h at 37°C ± LPS (100 ng/ml for THP-1 cells and 10 ng/ml for monocytes). Subsequently, the release of TNFα and IL-8 in supernatant samples was measured by enzyme-linked immunosorbent assay (ELISA) using Novex® Human Antibody Pair kits and following the manufacturer's protocol.

### THP-1 cell viability

Cell viability was assessed by measuring the proportion of THP-1 cells able to exclude propidium iodide (0.5 μg/ml) using the FACSCanto II flow cytometer (Becton Dickinson Biosciences, Franklin Lakes, NJ, USA).

### Assessment of differentiation of THP-1 cells to macrophage-like cells

A qualitative assessment of THP-1 cell morphology was carried out by imaging a minimum of 100 cells in three separate wells on a 6 well plate using a DMI3000 B inversion microscope (Leica, Heidelberg, Germany). The adherence of THP-1 cells to a 6 well plate after 72 h incubation was assessed by cell counts before and after detaching any adherent cells using a cell scraper. Furthermore, the expression of macrophage differentiation markers CD14 (CD14-PECy7, 561385, Becton Dickinson Biosciences), CD36 (CD36-PE, 336206, BioLegend, San Diego, CA, USA), and CD206 (CD206-PE, 321106, BioLegend) on the surface of THP-1 cells was measured using the BD FACSCanto-II flow cytometer (Becton Dickinson Biosciences). As a positive control THP-1 cells were differentiated into macrophage-like cells by incubation with 10 nM phorbol-12-myristate 13-acetate (PMA) for 72 h (22).

### MtDNA sequencing and bioinformatic analysis

This process was carried out as previously described by our group (23). Firstly, mtDNA was enriched using long-range polymerase chain reaction. In order to prevent contamination with nuclear-mitochondrial sequences, amplicons were polymerised using PrimeSTAR GXL DNA polymerase (error rate = 0.00108%, Takara Bio, Saint-Germain-en-Laye, France) in two overlapping fragments, using primer set-1: CCC TCT CTC CTA CTC CTG-F (m.6222–6239) and CAG GTG GTC AAG TAT TTA TGG–R (m.16133–16153), and set-2: CAT CTT GCC CTT CAT TAT TGC–F (m.15295–15315) and GGC AGG ATA GTT CAG ACG-R (7773–7791). Primer efficiency and specificity was assessed as successful after no amplification of DNA from ρ0 cell lines lacking mtDNA. Amplified products and DNA positive/negative controls were assessed by gel electrophoresis, and quantified using a Qubit 2.0 fluorimeter. Each amplicon was individually purified using Agencourt AMPure XP beads (Beckman-Coulter, USA), pooled in equimolar concentrations and re-quantified. For the mtDNA sequencing pooled amplicons were “tagmented,” amplified, cleaned, normalized, and pooled into 48 sample multiplexes using the Illumina Nextera XT DNA sample preparation kit (Illumina, CA, USA). The multiplex pools were sequenced using MiSeq Reagent Kit v3.0 (Illumina, CA, USA) in paired-end, 250 bp reads. Post run data, limited to reads with QV ≥ 30, were exported for analysis. Post-run FASTQ files were analyzed using an in-house developed bioinformatic pipeline. Reads were aligned to the revised Cambridge reference sequence (NC_012920) using BWA v0.7.10, invoking–mem (24). Aligned reads were sorted and indexed using Samtools v0.1.18 (25), duplicate reads were removed using Picard v1.85 (http://broadinstitute.github.io/picard/). Variant calling (including somatic calling) was performed in tandem using VarScan v2.3.8 (26, 27) (minimum depth = 1,500, supporting reads = 10, base-quality (BQ) ≥ 30, mapping quality (MQ) ≥ 20 and variant threshold = 1.0%), and LoFreq v0.6.1 (28). Concordance calling between VarScan and LoFreq was >99.5%. Concordant variants were annotated using ANNOVAR v529 (29) In-house Perl scripts were used to extract base/read quality data and coverage data.

### Assessment of mitochondrial mass and respiratory chain enzyme activity

The uptake of 2.5 μM nonyl acridine orange (NAO, a dye that localizes to cardiolipin on the mitochondrial inner membrane) by THP-1 cells over 30 min was determined by measuring fluorescence (excitation wavelength 488 nm, bandpass filter 530/30 nm) using the FACSCanto-II flow cytometer (30). As a positive control mitochondrial mass was increased in THP-1 cells by pre-incubation for 72 h in DMEM supplemented with 5 mM galactose and lacking glucose (31).

Citrate synthase is a nuclear DNA-encoded enzyme that catalyzes the initial reaction in the citric acid cycle in the mitochondrial matrix and provides a quantitative marker of cellular mitochondrial content (32). A spectrophotometric assessment of the activity of citrate synthase on THP-1 cell homogenate was carried out as previously described using the MultiSkan Ascent plate reader (33). Complex IV activity was determined by the rate of oxidation of reduced cytochrome C using the Complex IV Human Specific Microplate Assay Kit (Abcam Danvers, MA, USA).

### Phagocytosis of fluorescein-labeled *Escherichia coli*

Serum-opsonised fluorescein-labeled *Escherichia coli* K-12 strain were incubated with THP-1 cells at a multiplicity of infection of 10:1 for 1 h at 37°C. After washing and quenching extracellular fluorescence through the addition of 0.1% trypan blue (Sigma-Aldrich), the proportion of cells internalizing bacteria was then measured using the FACSCanto II flow cytometer (Becton Dickinson Biosciences, Franklin Lakes, NJ, USA).

### Mitochondrial bioenergetics assessment

Mitochondrial membrane potential was measured following incubation with 5 μM JC-1 (T3168) for 30 min the red (excitation wavelength 561 nm, bandpass filter 186/15 nm) and green (488 nm, 530/30 nm) fluorescence using the LSRFortessa X20 flow cytometer (Becton Dickinson Biosciences). As a positive control cells were pre-treated for 10 min with 100 nM valinomycin, a potassium-selective ionophore that dissipates the mitochondrial membrane potential (34). The production of ROS was determined by measuring the oxidation of 1 μM DCF-DA (D399). After 30 min the fluorescence (absorption wavelength 488 nm, band pass filter 530/30 nm) was measured on the BD FACSCanto-II flow cytometer (35). As a positive control cells were treated with 100 μM hydrogen peroxide.

Mitochondrial respiration and Extracellular acidification rate (ECAR) was determined using the Seahorse XF96^e^ Extracellular Flux analyser (both Seahorse Biosciences, Chickopee, MA, USA) as previously described (36). 0.8 × 10^5^ THP-1 cells were seeded in 175 μl of an assay medium per well, consisting of MEM supplemented with 11.1 mM D-Glucose and 2 mM L-Glutamine and adjusted to pH 7.0. Oxygen consumption rate (OCR) was measured at baseline and following the sequential addition of 1 μM oligomycin (a complex V inhibitor), 0.5 μM then 1 μM carbonyl cyanide 4-(trifluoromethoxy) phenylhydrazone (fCCP, an electron transport chain uncoupler) and finally 1 μM rotenone (a complex III inhibitor) plus 1 μM antimycin A (a complex I inhibitor). During each of the four stages of the assessment the OCR was measured in 16 wells per condition at 3 different time points. All OCR data were normalized to the total protein per well, which was determined using the Bradford assay.

### Nucleic acids extraction and quantification by real time PCR

For the determination of mtDNA copy number total DNA was extracted from cell pellets using the DNeasy blood and tissue kit (Qiagen, Valencia, CA, USA). The relative mtDNA copy number was determined by comparing the level of the mtDNA-encoded MT-ND1 gene (primers: F - ACGCCATAAAACTCTTCACCAAAG, R - GGGTTCATAGTAGAAGAGCGATGG) to that of the nuclear reference gene B2M (primers: F - CACTGAAAAAGATGAGTATGCC, R - AACATTCCCTGACAATCCC) by real-time quantitative polymerase chain reaction (qPCR) using the SYBR® Green technique and the MyiQTM PCR machine (both BioRad, Hercules, CA, USA) (37).

In the quantification of mRNA levels, RNA was extracted from pellets of 4 × 10^6^ THP-1 cells using the RNeasy mini kit (Qiagen) and single-stranded complementary DNA (cDNA) was synthesized using the High Capacity cDNA Reverse Transcription Kit. Following this the relative transcription of specific genes was determined by RTqPCR using the Taqman® Gene Expression Assay (β-ACTIN-Hs01060665_g1, GAPDH–Hs02758991_g1, HMOX1–Hs01110250_m1, SOD2–Hs00167309_m1) and the 7500 Fast Real Time PCR System. The relative amount of cDNA for each specific target was determined by comparison with the control housekeeping genes ACTB and GAPDH using the ΔCt method.

### Western blot

THP-1 cells were lysed using a lysis buffer containing 1% Triton X and the protease inhibitor phenylmethanesulfonyl fluoride (1 mM) (both Sigma-Aldrich) and the protein concentration in the lysates determined by Bradford assay. Equal amounts of protein were separated on the basis of size by sodium dodecyl sulfate polyacrylamide gel electrophoresis (SDS-PAGE), transferred onto polyvinylidene fluoride membranes and blotted with different antibodies, assessing the signal intensity after addition of an enhanced chemiluminescent substrate using the MultiSpectral Imaging System (UVP, Upland, CA, USA). The following antibodies were used; anti-mouse Ig-HRP (0260) and anti-rabbit-HRP (0448) from Dako (Cambridge, UK), β-actin (mouse, ab8226) and SOD2 (rabbit, ab13533) from Abcam (Danvers, MA, USA), LC3-I/II (rabbit, CS54995) from Cell Signalling (Beverley, MA, USA), Mitoprofile® Total OXPHOS antibody cocktail (mouse, MS604) from MitoSciences (Eugene, OR, USA) and TFAM (mouse, NBP1-71648) from Novus Biological (Cambridge, UK).

### Measurement of autophagy by detection of LC3-II by western blot–

Following a 2 h incubation in the presence or absence of 10 μM chloroquine (an inhibitor of autophagosome degradation) protein was extracted from THP-1 cells and the amount of LC3-II relative to the housekeeping protein β-actin was determined by Western blot.

### Confocal microscopy to assess mitophagy

Serum-starved THP-1 cells incubated in RPMI 1640 medium without FCS for 2 h were used as a positive control for the induction of mitophagy and treatment with 5 nM bafilomycin A1 for 2 h was used to prevent autophagosome turnover. The THP-1 cells were adhered to slides by cytospin, fixed using 4% paraformaldehyde, permeabilised using 0.1% Triton X, and blocked to prevent non-specific antibody binding using 2% bovine serum albumin. The slides were then incubated with the primary antibodies (OXPHOS complex II–mouse, 459200, ThermoFisher Scientific; LC3-II–rabbit, CS54995, Cell signaling) for 16 h at 4°C, washed and incubated with fluorochrome-conjugated secondary antibodies (anti-mouse IgG-Oregon Green–O6380, anti-rabbit IgG-Alexa Fluor 568–A11011) and the nuclear dye DAPI (D3571) for a further 2 h at room temperature. Co-localisation of LC3-II and mitochondrial complex II was determined using the SB2 UV confocal microscope and the X63 HCX PL APO lens (both Leica, Heidelberg, Germany). Each experimental condition was assessed in triplicate with images taken for a minimum of 100 cells over 3 separate fields of view for each slide. The images were analyzed using Volocity software (PerkinElmer, Waltham, MA, USA). Co-localisation of mitochondrial complex II and LC3-II was assessed using the Mander's M1 co-localisation co-efficient (38).

## Statistical analysis

All experiments were carried out on a minimum of three biological replicates. The Shapiro-Wilk test was used to determine the normality of the data. Normally distributed data are presented as mean ± standard deviation, and were analyzed using one-way analysis of variance (ANOVA) with Dunnett's *post-hoc* analysis or independent *t*-tests. Non-normal data are presented as median and interquartile range and were analyzed using the Kruskal-Wallis analysis of variance with Dunn's *post-hoc* analysis. A *p*-value of <0.05 was defined as the threshold for statistical significance.

## Results

### Pre-incubation with LPS produces an endotoxin tolerance phenotype in THP-1 cells

After an initial exposure to LPS (100 ng/ml) for 0–72 h the ability of THP-1 cells to respond to a second inflammatory stimulus was assessed. THP-1 cells pre-incubated with LPS for 2–48 h had a significantly reduced ability to release the pro-inflammatory cytokines tumor necrosis factor alpha (TNFα) and interleukin (IL)-8 in response to a second stimulation with LPS (100 ng/ml) (Figure 1B). Conversely, following pre-incubation with LPS the ability of THP-1 cells to phagocytose *Escherichia coli* was significantly enhanced (Figure 1C). There was no evidence that exposure to LPS adversely affected THP-1 cell viability (Figure 1D). Study of the morphology (Figure 1E), adherence capacity (Figure 1F) and cell surface markers (Figures 1G,H) confirmed the absence of differentiation into macrophage-like cells as compared with the positive control treated with PMA for 72 h. Macrophage polarization correlates with changes in metabolism (39). Similarly to the surface markers, measurement of the Extracellular acidification rate of the media, which is widely used as glycolysis surrogate (40) did not show any changes after LPS treatment (Figure 1I). These findings are consistent with a change in immune phenotype of THP-1 cells following treatment with LPS that is characteristic of the induction of endotoxin tolerance (41).

### Resolution of early oxidative stress following exposure of THP-1 cells to LPS

**Figure 2:**
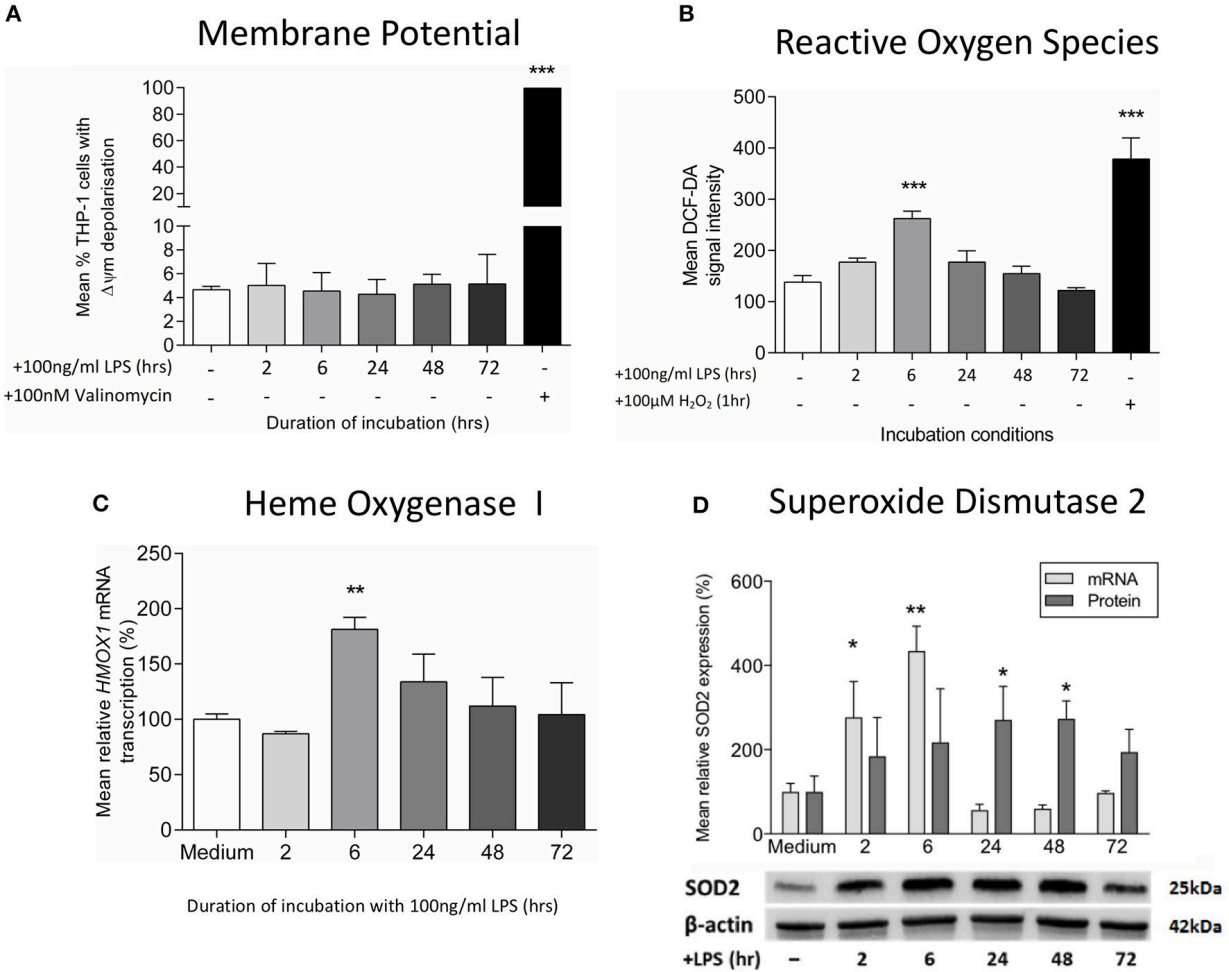
Exposure to LPS leads to early oxidative stress but no increase in mitochondrial membrane potential. Mitochondrial membrane potential (Δψm) and oxidative stress were assessed following incubation of THP-1 cells with LPS (100 ng/ml) for 0–72 h. **(A)** The proportion of THP-1 cells with Δψm was determined by measuring the JC1 fluorescence by flow cytometry (positive control−100 nM valinomycin) (*n* = 3). **(B)** Reactive oxygen species production was measured by oxidation of DCF-DA using flow cytometry (positive control−100 μM hydrogen peroxide, H_2_O_2_) (*n* = 3). **(C)** The mRNA transcription of *HMOX1* relative to *ACTB* and *GADPH* was determined by RTqPCR (*n* = 3). **(D)** The mRNA transcription (relative to *ACTB* and *GADPH*) and protein expression (relative to β-actin) of *SOD2* was measured using RTqPCR and Western blot (*n* = 3). Data are presented as mean ± standard deviation (mRNA and protein data are relative to the mean of the medium control); *p < 0.05, **p < 0.01, ***p < 0.001.

Next, we studied the effects of LPS treatment on mitochondrial dysfunction markers. We did not find any difference in the mitochondrial membrane potential on LPS-treated THP-1 cells (Figure 2A), confirming an absence of mitochondrial membrane depolarization, a process that reflects the leakage of proton across the inner mitochondrial membrane due to a loss of mitochondrial integrity (20). However, there was an early increase in reactive oxygen species production as measurement of hydrogen peroxide (Figure 2B), accompanied with higher mRNA levels of *heme oxygenase-1*, a gene that is up-regulated during oxidative stress (Figure 2C) (42). The resolution of this LPS-induced oxidative stress occurred in association with the stimulation of antioxidant defenses, as evidenced by increased levels of the mitochondrial antioxidant superoxide dismutase-2 (SOD2) (Figure 2D). Despite being particularly vulnerable to oxidative damage, we did not find any evidence of mtDNA deletions or mutations in LPS-exposed THP-1 cells (Supplementary Table 1). Thus, treatment of THP-1 cells with LPS leads to early oxidative stress, a process that is reversed by the induction of mitochondrial antioxidant defenses.

### An early induction of mitophagy following exposure of THP-1 cells to LPS

**Figure 3:**
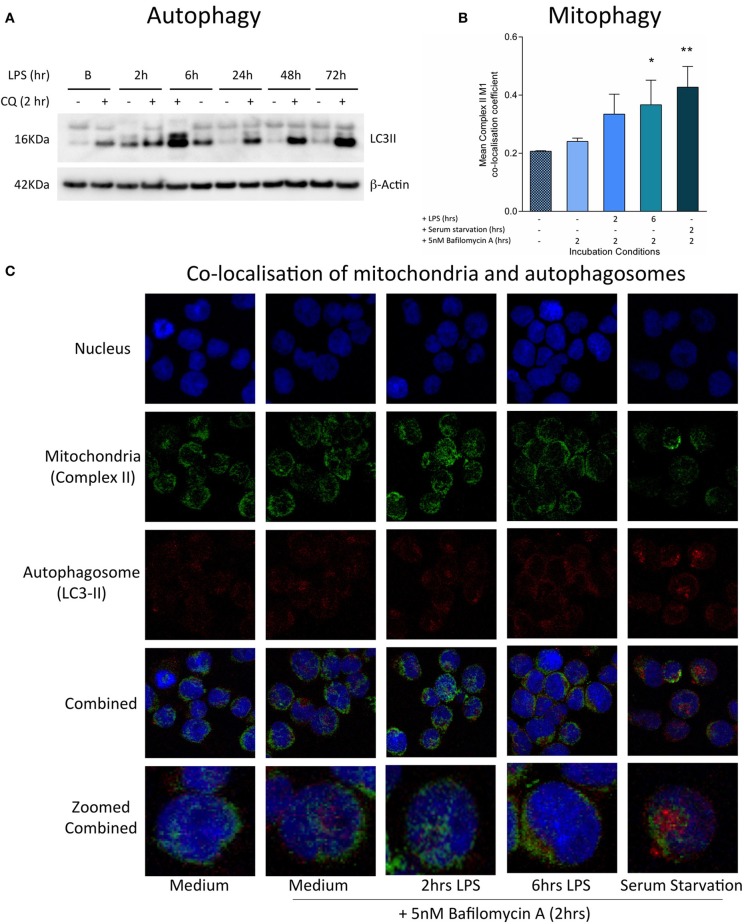
Induction of mitophagy in THP-1 cells following exposure to LPS. THP-1 cells were incubated with LPS (100 ng/ml) before measuring the levels of autophagy and mitophagy. **(A)** Autophagic flux was assessed by measuring the accumulation of LC3-II relative to β-actin using Western blot (*n* = 3). **(B)** Confocal microscopy provided a measure of mitophagy by measuring the co-localisation of mitochondrial complex II with the autophagosome marker LC3-II using the Mander's M1 co-localisation co-efficient (*n* = 4). **(C)** Representative confocal microscopy images with staining for the nucleus (blue), mitochondrial complex II (green), and LC3-II (red) after incubation of THP-1 cells with LPS (100 ng/ml) for 0–6 h. In all experiments serum starvation was used a positive control and accumulation of autophagosomes was facilitated by treatment with 10 μM chloroquine (CQ) or 5 nM bafilomycin A1 for the final 2 h. Data are presented as mean ± standard deviation; *p < 0.05, **p < 0.01.

Increased reactive oxygen species production is a feature of mitochondrial dysfunction, which has been found to activate mitophagy and target mitochondria for degradation (43). Consistent with this, treatment with LPS was found to stimulate both autophagy and mitophagy contemporaneously with the generation of oxidative stress, during the first hours of exposure. There was an early LPS-induced activation of autophagy, as indicated by significantly increased accumulation of the LC3-II protein, a constituent of the autophagosome, in THP-1 cells after treatment with LPS for 2–6 h (Figure 3A). The induction of mitophagy in LPS-treated THP-1 cells was then confirmed through the measurement of co-localization of mitochondria to autophagosomes using confocal microscopy (Figures 3B,C).

### Induction of mitochondrial biogenesis and increased mitochondrial respiration in LPS-exposed THP-1 cells

**Figure 4:**
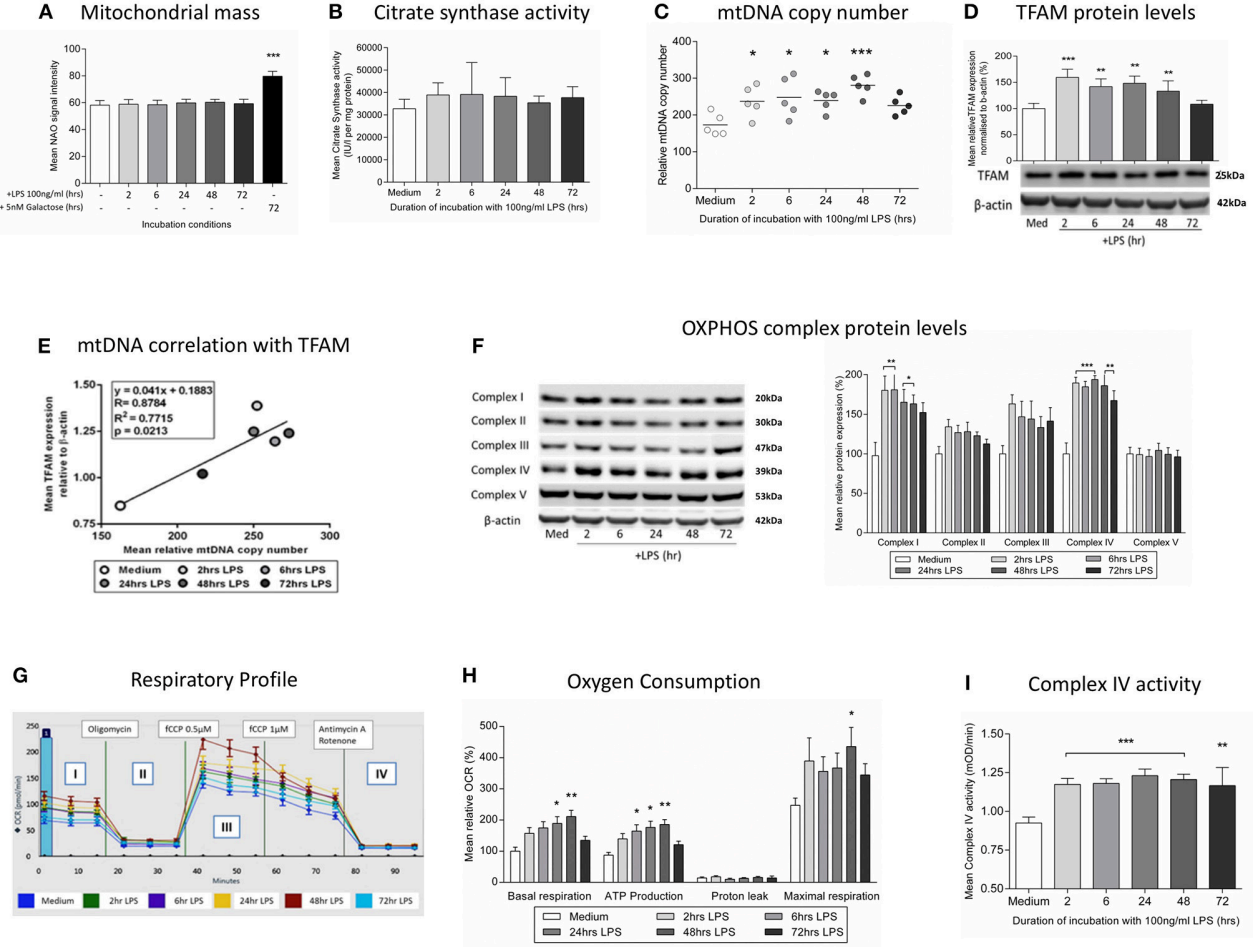
Induction of mitochondrial biogenesis following exposure of THP-1 cells to LPS. THP-1 cells were incubated with LPS (100 ng/ml) for 0–72 h before assessing mitochondrial biogenesis and respiration. **(A)** Mitochondrial mass was assessed by measuring the uptake of NAO using flow cytometry, (positive control - glucose-free medium supplemented with 5 mM galactose) (*n* = 4). **(B)** A colorimetric assay was used to assess the activity of the mitochondrial matrix enzyme citrate synthase (*n* = 3). **(C)** mtDNA copy number was determined by measuring *MT-ND1* relative to *B2M* using qPCR (*n* = 5). **(D)** The level of TFAM protein relative to β-actin was determined by Western blot (*n* = 4). **(E)** Scatter plot, linear regression and Pearson's correlation of the relationship between mtDNA copy number and TFAM protein levels. *p < 0.05, **p < 0.01, ***p < 0.001. **(F)** Representative images of protein bands for subunits of the five mitochondrial OXPHOS complexes on PVDF membrane following Western blot and quantification of the subunits of the five mitochondrial OXPHOS complexes relative to β-actin (*n* = 4). **(G)** Representative example of the respiratory profile and **(H)** oxygen consumption rate for different aspects of mitochondrial respiration following exposure to LPS (100 ng/ml) for 0–72 h as determined using the Seahorse XF96^e^ extracellular flux analyser (*n* = 5). **(I)** A colorimetric assay was used to measure the activity of OXPHOS complex IV(*n* = 3). The data are represented as **(A)** individual values with a line indicating the mean or **(B,C,F,H,I)** mean ± standard deviation relative to the mean in the medium control; *p < 0.05, **p < 0.01, ***p < 0.001.

We next studied whether the induction of mitophagy after treatment with LPS was associated with changes in the overall mitochondrial mass. We did not observe changes in the level of the internal membrane marker cardiolipin (Figure 4A) or the matrix marker citrate synthase activity (Figure 4B), suggesting that there must be a parallel replacement of any degraded mitochondria. This was confirmed by assessments indicating that there was an early and sustained activation of mitochondrial biogenesis in THP-1 cells after LPS treatment. We observed a significant increase in THP-1 cell mtDNA copy number in THP-1 cells treated with LPS for 2–48 h (Figure 4C), that significantly correlated with the level of mitochondrial transcription factor A (TFAM), a key regulator of mitochondrial biogenesis that is bound to mtDNA (Figures 4D,E). Furthermore, LPS-treated THP-1 cells had a significant increase in protein levels for constituents of the inner mitochondrial membrane OXPHOS complexes I and IV, the two complexes containing the greatest number of mtDNA-encoded proteins (Figure 4F) (44). These results suggest that there is a coordinated up-regulation of mitochondrial biogenesis and mitophagy, leading to the maintenance of mitochondrial mass in THP-1 cells following an inflammatory insult in the form of LPS treatment.

The overall functional consequences of the increases in mitochondrial biogenesis and mitophagy were assessed by measurement of mitochondrial oxygen consumption. It was shown that exposure to LPS resulted in increased basal mitochondrial respiration and mitochondrial ATP production with no effect on non-mitochondrial oxygen consumption (Figures 4G,H), findings consistent with the increased levels of OXPHOS complexes I and IV (Figure 4F) and the increased activity of isolated complex IV (Figure 4I). Thus, in association with a shift to an endotoxin tolerance phenotype, THP-1 cells exposed to LPS have early evidence of oxidative stress which resolves in association with the induction of mitophagy and mitochondrial biogenesis, resulting in maintained cell viability and increased mitochondrial respiration.

### Induction of endotoxin tolerance and mitochondrial biogenesis in human monocytes treated with LPS

**Figure 5:**
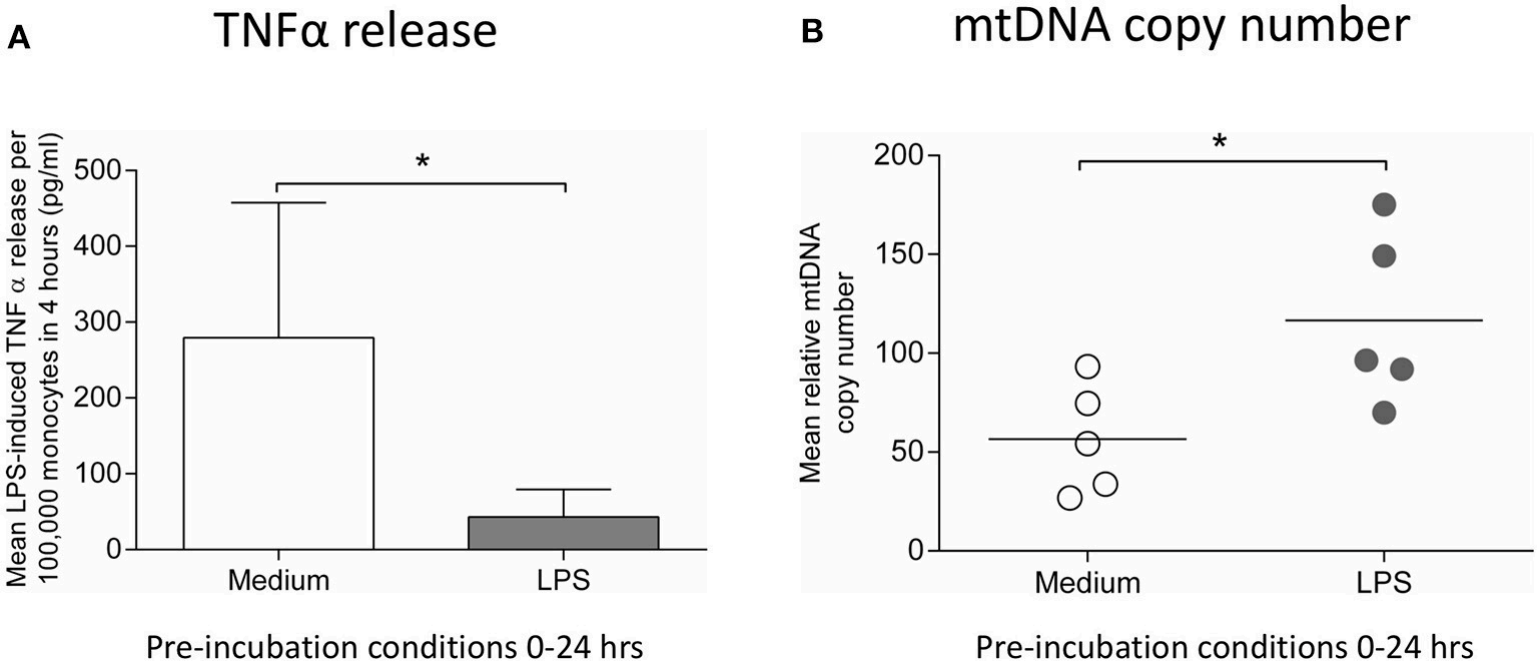
Incubation of monocytes with LPS for 24 h results in reduced ability to release TNFα in response to a second LPS stimulus and increased mtDNA copy number. Blood monocytes were incubated with LPS (10 ng/ml) before measuring cytokine release and mtDNA copy number. **(A)** The release of TNFα in response to a second 4 h exposure to LPS (10 ng/ml) was measured by ELISA (*n* = 5). **(B)** mtDNA copy number was determined by measuring *MT-ND1* relative to *B2M* using qPCR (*n* = 5). Data are represented as **(A)** mean ± standard deviation or **(B)** individual values with a line representing the mean. *p < 0.05 on independent *t*-test.

To confirm the relevance of the findings in THP-1 cells, human monocytes were pre-incubated with medium or 10 ng/ml LPS for 24 h before measuring cytokine release and mtDNA copy number. The induction of endotoxin tolerance was indicated by the finding that monocytes pre-incubated with LPS for 24 h had a significantly reduced ability to release TNFα release in response to a second exposure to LPS (Figure 5A). There was also a significant increase in mtDNA copy number in those monocytes treated with LPS, suggesting a similar induction of mitochondrial biogenesis to that seen in THP-1 cells (Figure 5B).

## Discussion

The regulation of the compensatory pro-survival cellular mechanisms that are triggered following an inflammatory insult and the mechanisms by which they become deranged during sepsis are not well understood. We have used an endotoxin tolerance model to induce a temporary state of immune deactivation in human monocytic cells resembling that seen in septic monocytes (18). This shift to an anti-inflammatory phenotype after exposure to LPS was found to occur in parallel with the activation of responses aimed at maintaining mitochondrial homeostasis; namely antioxidant defenses, mitophagy, and mitochondrial biogenesis. In this model the resultant selective replacement of dysfunctional mitochondria leads to an increase in overall mitochondrial efficiency, as evidenced by increased mitochondrial respiration despite unchanged mitochondrial mass, in association with the resolution of oxidative stress and the recovery of pro-inflammatory cytokine release by 72 h.

Mitophagy involves the lysosomal degradation of mitochondria following encapsulation in an autophagosome (45). This process is key for the maintenance of quality control because damaged mitochondria are specifically targeted for destruction (46). Although data from human studies are very limited, it appears that mitophagy may be insufficient during severe sepsis resulting in persistence of dysfunctional mitochondria that can lead to deficient respiration (47, 48). Moreover, animal sepsis models suggest that mitophagy is an important component of the recovery mechanisms, with inadequate mitochondrial degradation associated with increased organ damage, persistence of oxidative stress, and exacerbation of inflammation through increased inflammasome formation (49–51). In keeping with this, reports in cell culture and murine models indicate that autophagy and mitophagy are stimulated following ligand binding to pattern recognition receptors in a process which is implicated in suppressing pro-inflammatory cytokine release and oxidative stress through inhibition of the nucleotide-binding domain, leucine-rich-containing family, pyrin domain containing-3 (NLRP3) inflammasome (52, 53).

Mitochondrial biogenesis is a dynamic process during which pre-existing mitochondria grow and divide in response to physiological conditions or cellular energy requirements (54). It is controlled by a complex network of hormones and signaling pathways which regulate the expression of mitochondrial transcription factors that in turn co-ordinate the expression of mitochondrial genes in the nuclear and mitochondrial genomes (55). Mitochondrial biogenesis appears to be an essential response that is required to compensate for the adverse effects of inflammation on mitochondrial structure and function (56). In a wide variety of animal models and a few small observational human studies there is increasing evidence that expanding the mitochondrial population promotes the recovery from sepsis (57–59). The effects of this inflammation-induced mitochondrial biogenesis may actually extend beyond maintaining homeostasis and facilitate an increase in overall cellular respiration, as seen in our model, potentially leading to an increased resistance to the negative effects of excessive inflammation (36, 60).

Although human data have been lacking, animal models suggest that there is an integrated up-regulation of mitophagy and mitochondrial biogenesis following an inflammatory insult (43). Both processes appear to be directly triggered following the recognition of inflammatory stimuli; inhibition of Toll-like receptor-4 (TLR-4, the main pattern recognition receptor for LPS-induced signaling) after caecal ligation and puncture or LPS treatment prevents the activation of both mitophagy and mitochondrial biogenesis (48, 61). Mitochondrial turnover may be integrated with antioxidant defenses and anti-inflammatory responses through the activation of redox-sensitive signaling pathways (62). Murine models suggest that during sepsis the inducible antioxidant enzyme heme oxygenase-1 stimulates the expression of nuclear factor (erythroid-derived-2)-like 2 (Nrf2), a transcription factor that binds to anti-oxidant response elements on gene promoters for transcription factors regulating mitochondrial biogenesis, mitophagy, and anti-inflammatory responses (63, 64). Nrf2^−/−^ knockout mice have impaired ability to upregulate these processes, leading to more severe sepsis (43, 65). Alternatively, a group of deacetylases termed silent information regulators (sirtuins), with activity dependent on the presence of the oxidized form of the respiratory chain enzyme nicotinamide adenine dinucleotide (NAD+), facilitate responses to alterations in cellular energy levels and may thus link metabolism with immunity (66). In the nucleus SIRT1 activates both mitochondrial biogenesis and autophagy, but is also involved in resolution of inflammation through the negative regulation of pro-inflammatory pathways (67, 68). In the mitochondria SIRT3 is essential for effective mitochondrial biogenesis and can increase OXPHOS activity and activate antioxidant responses (69). In one study, following exposure to LPS, the sequential activation of SIRT1 and SIRT3 was found to integrate the induction of mitochondrial biogenesis with the down-regulation of pro-inflammatory responses (36). Another potential link between immunity and mitochondrial homeostasis is suggested by findings that the intracellular chaperone heat shock protein-90 (HSP90) is both essential for effective clearance of defective mitochondria by mitophagy and implicated, through accumulation at the cell surface, in the suppression of TNFα production in response to LPS (70, 71).

To our knowledge we have shown for the first time that mitochondrial biogenesis and mitophagy are important in the responses of immunologically relevant human cells to an inflammatory insult, providing important insights into the compensatory mechanisms that may go wrong during sepsis. However, it should be noted that this study has some limitations. Firstly, our model predominantly used THP-1 cells because human monocytes have considerable inter-individual variability, a limited life span, a propensity to rapidly differentiate *in vitro*, and are difficult to isolate in large numbers (72, 73). THP-1 cells have similar morphology, surface antigens and secretory products to blood monocytes and have been used extensively to study monocyte and macrophage functions (74, 75). However, they represent a simplified model and have important differences to human monocytes that are particularly relevant when assessing the response to LPS, including significantly reduced expression of the LPS receptor CD14 and altered cytokine production (76, 77). In view of this, while we have detected similar effects of LPS exposure on TNFα release and mtDNA copy number in monocytes, our results are only suggestive of responses that may occur in human monocytes and an *in vivo* validation, for example in a human LPS challenge model, is ultimately required (78).

It should also be noted that, while we have observed an immune phenotype consistent with endotoxin tolerance, there are other potential explanations for the decrease in pro-inflammatory cytokine release following prior exposure to LPS that have not been fully explored. These include the presence of LPS-binding protein, a down-regulation in TLR-4 expression or a failure of internalization of TLR-4-LPS complexes (79, 80). Similarly, although we have found no evidence that LPS exposure leads to THP-1 cell macrophage-like differentiation, M2 polarization remains a potential mechanism for the change in immune phenotype and mitochondrial respiration seen in this study (81, 82). In addition, endotoxin tolerance is not an ideal model of sepsis as a single, sterile stimulus does not mimic the overwhelming, multiple, and persistent inflammatory triggers seen in sepsis. Rather, by using endotoxin tolerance, we are likely to mimic the processes occurring when an infection is successfully cleared instead of a situation in which sepsis is triggered. Furthermore, the dose of LPS required to induce endotoxin tolerance in this study (100 ng/ml) is significantly higher than the concentration of LPS that has been measured in the serum of patients with septic shock (83). Finally, we have not elucidated the mechanisms underlying our observations, which means that causality and the precise relationships between the changes observed remain to be determined.

In clinical studies and animal models survival and recovery of cellular functions in sepsis appear to be dependent on the induction of compensatory responses, including those aimed at preventing excessive inflammation and those that maintain mitochondrial homeostasis through the activation of mitochondrial biogenesis, mitophagy and antioxidant defenses. Here we show for the first time in human monocyte-like cells that there is a contemporaneous, co-ordinated upregulation of mitochondrial biogenesis, mitophagy and antioxidant defenses during endotoxin tolerance, which results in the resolution of oxidative stress and increased mitochondrial respiratory activity. This provides important insights into the relationship between mitochondria and the innate immune response, as well as the coordination of compensatory responses that are required to maintain cell viability and function following an inflammatory insult. We speculate that these processes may go awry when the insult leads to sepsis.

## Author contributions

JW, AG-D, SB, AR, PL PC, and AS formulated the research hypothesis and designed the experiments. JW, AG-D, AP, M-HR-S, and JS carried out the experimental procedures and data analysis, assisted with data collection, and interpretation. The manuscript was prepared by JW, AG-D, AS, and PC.

### Conflict of interest statement

The authors declare that the research was conducted in the absence of any commercial or financial relationships that could be construed as a potential conflict of interest.
