# Supplementary material for: Exposure of Monocytic Cells to Lipopolysaccharide Induces Coordinated Endotoxin Tolerance, Mitochondrial Biogenesis, Mitophagy, and Antioxidant Defenses
Source: Front Immunol. 2018 Sep 27;9:2217. doi: 10.3389/fimmu.2018.02217 (PMC6170658; doi:10.3389/fimmu.2018.02217)
Supplement: Supplementary file 1 [file Table_1.DOCX]

**SUPPLEMENTARY TABLE**

|  |  |  | ***Med_A*** | ***Med_B*** | ***2hr_A*** | ***2hr_B*** | ***6hr_A*** | ***6hr_B*** | ***24hr_A*** | ***24hr_B*** | ***48hr_A*** | ***48hr_B*** | ***72hr_A*** | ***72hr_B*** |
| --- | --- | --- | --- | --- | --- | --- | --- | --- | --- | --- | --- | --- | --- | --- |
| ***rCRS (bp)*** | ***Ref*** | ***VarAllele*** | ***Plasmy*** | ***Plasmy*** | ***Plasmy*** | ***Plasmy*** | ***Plasmy*** | ***Plasmy*** | ***Plasmy*** | ***Plasmy*** | ***Plasmy*** | ***Plasmy*** | ***Plasmy*** | ***Plasmy*** |
| 152 | T | C | 99.9 | 99.9 | 99.9 | 99.8 | 99.9 | 99.9 | 99.6 | 99.8 | 100.0 | 99.9 | 99.9 | 99.9 |
| 515 | A | G | 38.8 | - | 45.9 | 34.7 | - | 34.5 | - | 37.3 | - | 42.6 | 41.6 | - |
| 2716 | G | A | 2.8 | 2.8 | 3.0 | 2.4 | 2.6 | 3.0 | 2.8 | 2.6 | 2.7 | 2.8 | 2.8 | 3.0 |
| 4563 | G | A | 8.6 | 9.1 | 8.0 | 8.0 | 8.1 | 7.9 | 7.9 | 8.3 | 8.1 | 7.5 | 9.1 | 8.8 |
| 11740 | C | A | 6.6 | 6.0 | 6.6 | 6.1 | 6.4 | 6.0 | 6.3 | 6.0 | 6.5 | 6.1 | 6.6 | 7.1 |
| 13810 | G | A | 5.4 | 4.8 | 4.8 | 4.2 | 5.1 | 4.6 | 5.1 | 4.4 | 4.7 | 5.3 | 4.7 | 5.1 |
| 14233 | A | G | 19.7 | 19.8 | 19.0 | 18.5 | 19.7 | 19.5 | 20.0 | 18.6 | 21.4 | 19.2 | 18.8 | 19.2 |
| 15173 | G | A | 21.2 | 21.8 | 21.2 | 20.4 | 21.2 | 20.9 | 23.1 | 21.6 | 20.7 | 21.1 | 21.1 | 20.9 |
| 15975 | C | T | 2.2 | 1.9 | 2.0 | 2.1 | 2.3 | 2.3 | 2.0 | 1.7 | 2.6 | 2.0 | 2.2 | 2.1 |
| 16182 | A | C | 39.9 | - | 42.1 | 39.6 | - | 40.4 | - | 39.2 | - | 38.6 | 41.6 | 41.2 |
| 16311 | T | C | 99.9 | 99.9 | 100.0 | 99.9 | 99.8 | 99.9 | 99.8 | 99.9 | 99.9 | 99.9 | 99.9 | 99.9 |
| 16362 | T | C | 99.9 | 99.9 | 100.0 | 99.9 | 99.8 | 99.9 | 99.8 | 100.0 | 100.0 | 100.0 | 100.0 | 100.0 |
| 16519 | T | C | 100.0 | 99.9 | 100.0 | 99.9 | 100.0 | 99.9 | 100.0 | 99.9 | 100.0 | 100.0 | 100.0 | 99.9 |

***Supplementary Table 1 – Exposure to LPS does not lead to significant mutations and deletions in THP-1 cell mtDNA*.** THP-1 cells were incubated with LPS (100ng/ml) for 0-72 hours and mtDNA sequencing carried out prior to assessing heteroplasmy levels. Data represent the percentage of mtDNA transcripts with the listed variant allele.
